# Expansion of the ADOR Strategy for the Synthesis of Zeolites: The Synthesis of IPC‐12 from Zeolite UOV

**DOI:** 10.1002/anie.201700590

**Published:** 2017-03-13

**Authors:** Valeryia Kasneryk, Mariya Shamzhy, Maksym Opanasenko, Paul S. Wheatley, Samuel A. Morris, Samantha E. Russell, Alvaro Mayoral, Michal Trachta, Jiří Čejka, Russell E. Morris

**Affiliations:** ^1^ School of ChemistryUniversity of St AndrewsPurdie BuildingSt AndrewsKY16 9STUK; ^2^ J. Heyrovský Institute of Physical ChemistryAcademy of Sciences of the Czech Republicv.v.i., Dolejškova 3182 23Prague 8Czech Republic; ^3^ Department of Physical and Macromolecular ChemistryFaculty of ScienceCharles UniversityHlavova 8128 43Prague 2Czech Republic; ^4^ Advanced Microscopy Laboratory (LMA)Nanoscience Institute of Aragon (INA)University of ZaragozaMariano Esquillor, Edificio I+DZaragoza50018Spain; ^5^ Institute of Organic Chemistry and BiochemistryAcademy of Sciences of the Czech Republicv.v.i., Flemingovo nám. 216610PragueCzech Republic

**Keywords:** ADOR, germanosilicates, isoreticular materials, structure rearrangement, zeolites

## Abstract

The assembly–disassembly–organization–reassembly (ADOR) process has been used to disassemble a parent zeolite with the **UOV** structure type and then reassemble the resulting layers into a novel structure, IPC‐12. The structure of the material has previously been predicted computationally and confirmed in our experiments using X‐ray diffraction and atomic resolution STEM‐HAADF electron microscopy. This is the first successful application of the ADOR process to a material with porous layers.

For more than 60 years, zeolites have been almost exclusively prepared via hydrothermal,^1^ solvothermal,^2^ and ionothermal^3^ synthesis techniques. The recently discovered ADOR (assembly–disassembly–organization–reassembly) strategy^4^ is an alternative way to prepare new zeolite structures. The method consists of the chemically selective disassembly of a parent zeolite into its constituent layers. This is followed by organization of these units into a suitable relative orientation and the reassembly of the units into new materials. Controlled disassembly of the parent zeolite is possible when there is a weakness engineered into the structure during the initial synthesis.^5^ In general, this involves the regioselective substitution of silicon for germanium, which is much more hydrolytically sensitive than the silicon species, allowing selective dissolution of the germanium out of the material.

The ADOR process is fundamentally different from hydrothermal synthesis in that the final framework‐forming step is an irreversible condensation at high temperature (500–700 °C) rather than a reversible crystallization step. This leads to new zeolites that have unusual properties that include the possibility of preparing isoreticular families of materials with continuously controllable porosity.^6^ Of potential great importance is the possibility of preparing materials that do not obey the energy‐density rules^7^ associated with hydrothermal synthesis, leading to new zeolites that in the past would have been thought unfeasible synthetic targets.^8^ An important point to note is that the reassembly process is very easy to model, leading to final products that are computationally predictable,^9^ something that is very difficult in hydrothermal synthesis.

One crucial issue of the ADOR approach not yet demonstrated is its general applicability. Up to now, the ADOR approach to new materials has concentrated on the use of zeolite **UTL** as the parent material, although other parent zeolites, such as **IWW** have been successfully disassembled.^10^ Germanosilicate **UTL** is an ideal ADOR starting point because of its chemical composition and because of the stability of the layered units that are formed on disassembly. Herein we report the synthesis of a new zeolite, which we name IPC‐12, using the ADOR transformation of a germanosilicate parent zeolite with the **UOV** topology.^11^ We have previously predicted that **UOV** would be a good target,^12^ but since it has pores in three dimensions, rather than only in two as is the case for **UTL**, we could not rule out the possibility that the layers (which are porous) would be less stable than in **UTL**. In designing this successful procedure, particular attention has to be given to the factors controlling the ADOR process, such as appropriate chemical and structural properties of the parent material, optimized conditions for disassembly and reassembly of formed intermediates.

IPC‐12 retains the same pore systems as **UOV** in one direction (viewed perpendicular to the *bc* crystallographic plane (see Figure 1), but has new structural features when viewed in the other two directions. The structure of the new material has been confirmed using X‐ray diffraction and atomic resolution STEM‐HAADF electron microscopy.

**Figure 1:**
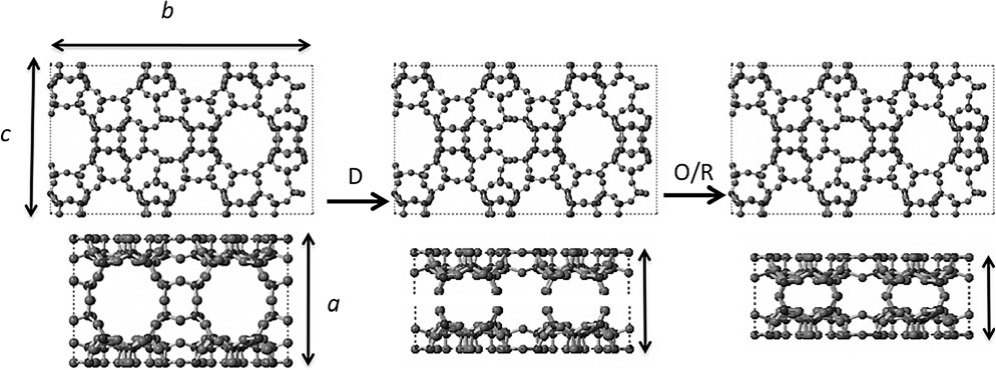
The predicted ADOR process starting with the disassembly (D) of a parent **UOV** zeolite into layered intermediates by removal of the d4r units, followed by the organization and reassembly steps (O/R) into the final material. Note that the process should not affect the structure of the layers themselves (as is seen in the top view), which means the intralayer unit cell parameters (*b* and *c*) remain constant but the interlayer unit cell parameter (*a*) decreases throughout the process.

It was previously predicted that different zeolites are potentially suitable for ADOR application on the basis of their topologies, and in particular the presence of double four ring (d4r) units in their structures.^4^, ^13^ Germanium is well known to site preferentially in the d4r units in many structures.^5^ The predicted transformations for the **UOV** parent structure are shown in Figure 1.

A sample of germanosilicate zeolite with the **UOV** topology was synthesized as described in the experimental section. The obtained sample was single phase and highly crystalline by X‐ray diffraction. The molar ratio of silicon to germanium used in the reaction mixture was 0.5, but chemical analysis (ICP) of the final UOV product indicated a Si/Ge ratio of 3.1. The use of ^19^F NMR spectroscopy after postsynthetic fluorination according to the method of Tuel and co‐workers^14^ revealed two main resonances (Supporting Information, Figure SI‐2). One peak at around −10 ppm is typically assigned to F^−^ occluded in Ge_4_Si_4_ d4rs^14^ and a resonance at −30 ppm that is typically assigned to F^−^ located in the siliceous layer surrounded only by silicon atoms. These results indicate that, as in the case of germanosilicate **UTL**, the germanium is preferentially accommodated into the d4r units between the layers, indicating that not only do the prepared germanosilicate **UOV** materials have a suitable topology for the ADOR process, but also a suitable chemical composition.

The next stage in the ADOR process is to complete the disassembly of the parent material into its layered components. The disassembly of the parent germanosilicate **UOV** was accomplished by exposure of the parent solid to acidic solutions of 0.1 m or 12 m HCl for 24 hours at room temperature. Both reactions gave the same product after calcination at 550 °C. The ADOR process can be followed by XRD as is shown in Figure 2. The direct way to assess the changes that occur during this process is the evaluation of the intensity and position of the peaks with *hkl* indexes related to inter‐ (*h*≠0) and intralayer (*h*=0) planes. In this regard, the 013 and 004 peaks retain their positions, as the *b* and *c* unit cell dimensions are not affected by the ADOR transformation (Figure 1), while 100, 111, and 102 are expected to change position markedly if disassembly of the initial zeolite takes place. In the case of **UOV**, the shift of the 100 to higher 2*θ* values (smaller unit cell *a* dimension) is particularly revealing of the structural changes (Figure 2). This confirms the prediction shown in Figure 1 of the shortening of the crystallographic *a* axis as the d4r units are removed from between the layers.

**Figure 2:**
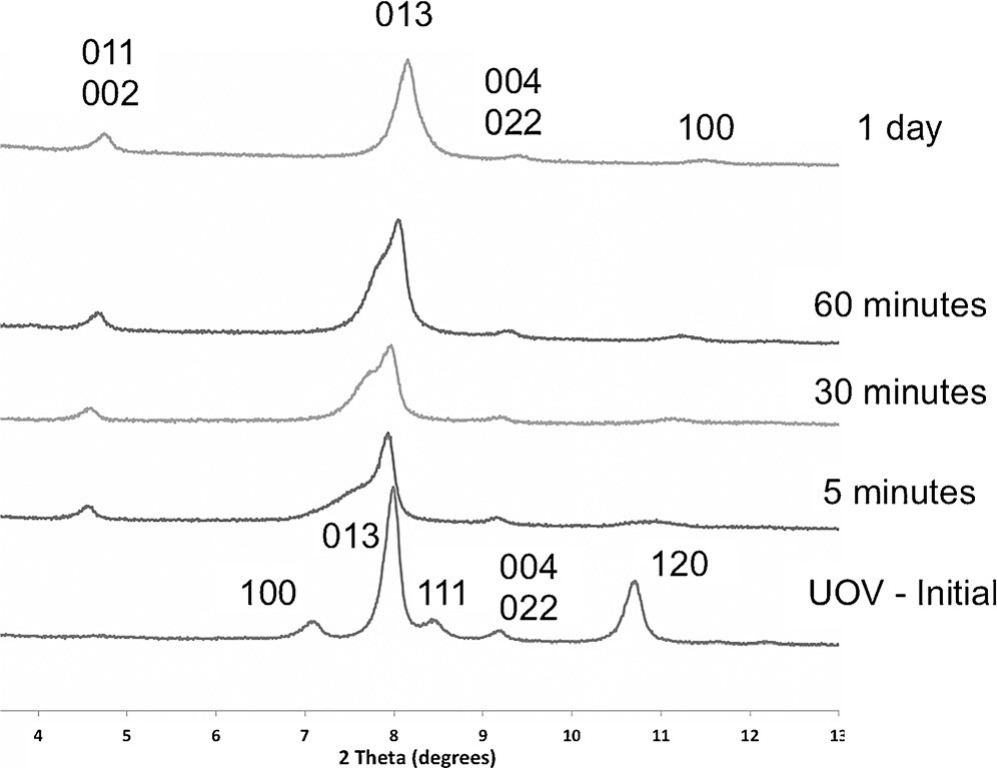
The XRD patters of the initial **UOV** and intermediates recovered after 5, 30, and 60 minutes of hydrolysis in 12 m HCl, together with the final material after treatment for 1 day. It is clear that the positions of those reflections with *h*=0 are approximately invariant during the process while those with *h*≠0 are significantly shifted, which is consistent with the predicted ADOR process for **UOV** shown in Figure 1.

The ADOR behavior of **UOV** differs from that of **UTL**, as the latter provides two different materials, while for **UOV** only one material is formed under these conditions. In the **UTL** ADOR process at low acidity the disassembly process dominates, but at very low pH (high acidity) the disassembly also happens quickly but is followed by a subsequent rearrangement process, with extra silicon‐containing bridges forming between the layers, resulting, after calcination, in a different zeolite IPC‐2.^15^, ^16^, ^17^ This rearrangement process does not occur in **UOV**, instead the higher acidity promotes reconnection of the layers without the intercalation of any extra silicon. The reconnection of the UOV‐derived layers is supported because they cannot be swollen using standard techniques, unlike the UTL‐derived layers, which can be swollen when first formed. The reasons why **UTL**‐ and **UOV**‐type zeolites behave so differently is yet to be discovered, but one must remember that the ADOR process is a subtle balance between several different processes (disassembly, organization, intercalation of species between the layers and reconnection of the layers) and it is not surprising that any one of these processes may be slower or thermodynamically disfavored in certain materials. This seems to be the case for **UOV** as the re‐intercalation step seen for **UTL** does not seem to occur.

The XRD patterns for the IPC‐12 materials correspond to each other and match well with theoretically predicted one. Given this information, and the fact that we have a predicted structure from computational work, Rietveld refinement against synchrotron X‐ray diffraction data was attempted. Compared to many very highly crystalline solids there is not as much data in the diffraction patterns for IPC‐12. This is not unusual for ADOR‐derived materials as any mistakes in the bonding formed in the irreversible final framework forming step cannot be healed as they can in the reversible crystallization of hydrothermal synthesis. This means that the successful Rietveld refinement requires rather heavy restraints on the Si−O and O−O interatomic distances. The fit observed and calculated diffraction patterns (Figure 3) is however, acceptable and the final structural model matches well to that predicted from the previous computational work.

**Figure 3:**
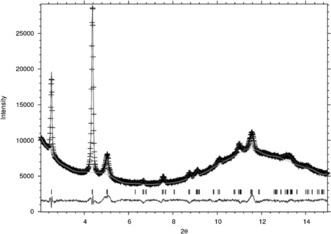
Final plot showing the observed (experimental) synchrotron X‐ray diffraction data (crosses), and the calculated XRD pattern (solid line) from the final Rietveld refinement, together with the difference between the two. Tick marks indicate the position of Bragg reflections.

The porous system of the parent **UOV** zeolite can be described as a combination of parallel 12‐ and 8‐ring channels going through the layers and “interlayer” 10‐ring channels. The 12 ring channels are arranged in a pseudo hexagonal arrangement, six such channels surrounding one of the 8‐ring channels. In the prediction for the ADOR transformation of **UOV** the hexagonal arrangement of the 12‐ and 8‐ring channels should remain unchanged as these layers should remain intact, and the product IPC‐12 should have exactly the same pseudo hexagonal arrangement. This structural model of IPC‐12 was confirmed by atomic resolution spherical aberration (*C_s_*) corrected STEM‐HAADF images analysis. As is clearly seen in Figure 4, the pseudohexagonal arrangement of the 12‐ring channels predicted by the XRD model (Figure 4 a) is clearly visible in the STEM‐HAADF images (Figure 4 b).

**Figure 4:**
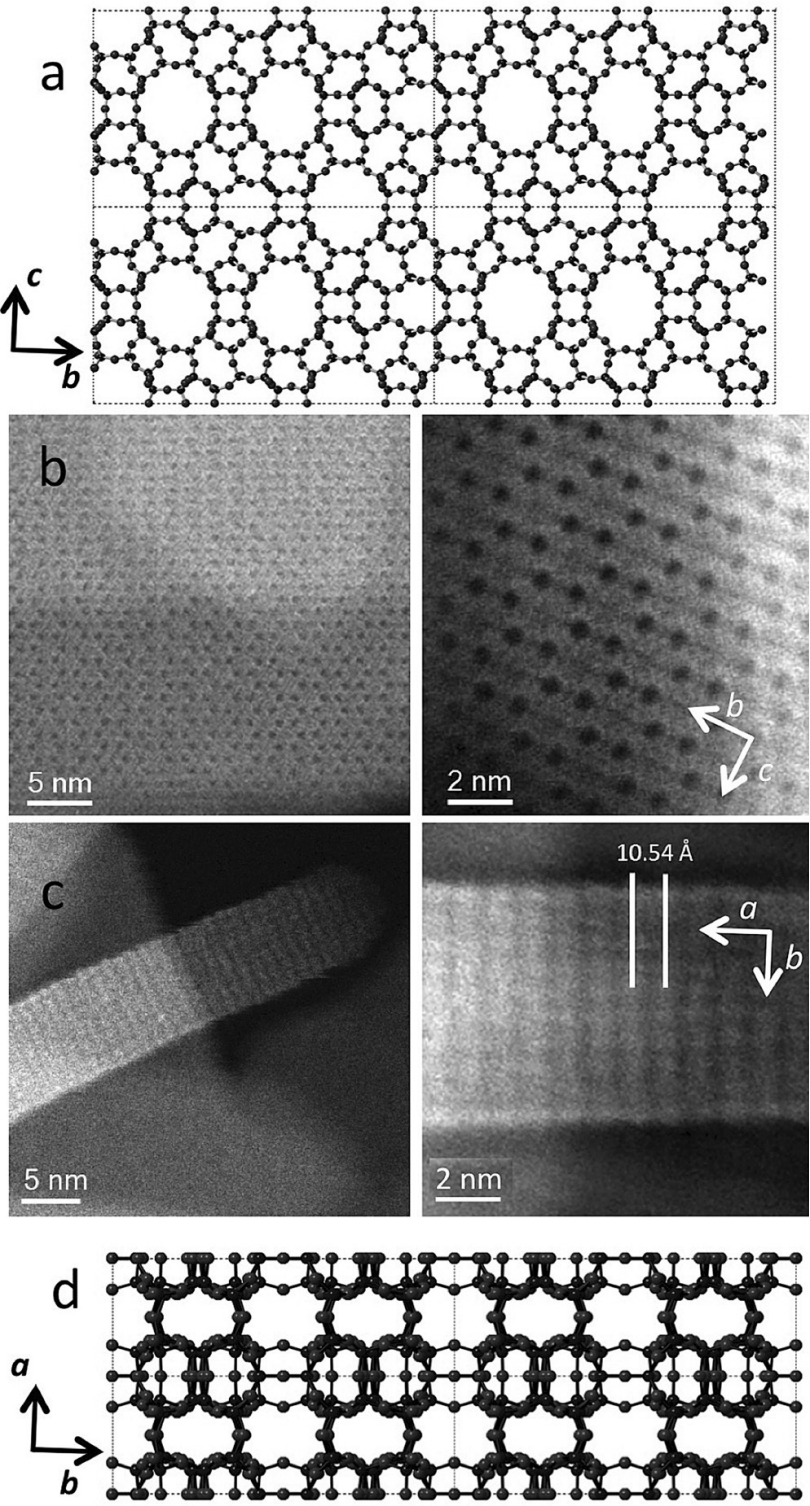
a) A view of the crystallographic model of IPC‐12 viewed in the *ab* projection, showing the pseudo hexagonal arrangement of 12‐ring channels b) Atomic resolution spherical aberration (*C_s_*) corrected STEM‐HAADF images of IPC‐12 in the same *bc* projection as (a) illustrating the presence of the same arrangement of 12‐ring channels in the real material. c) STEM‐HAADF image of IPC‐12 viewed in the *ab* projection showing the arrangement of layers in the final material, with an interlayer separation of about 10.5 Å (d). A view of the crystallographic model in the same *ab* projection showing the same arrangement of layers as in (c).

The major difference between the **UOV** and IPC‐12 topologies is the connectivity perpendicular to these 12‐/8‐ring channels. In the parent UOV there is a 10‐ring channel in this direction. However, in IPC‐12 the prediction is that there should be no channel structure in this direction, the 10‐rings being reduced to 6‐rings by loss of the d4r units. Again this model is confirmed by both the XRD and STEM‐HAADF. Figure 4 c shows the images of the *ac* projection of the IPC‐12 structure, showing no obvious channels and a repeat distance of about 10.5 Å (Figure 4 c), which is consistent with the layers in the structure now being connected by 6‐rings, as predicted from the Rietveld refinement (Figure 4 d).

Taking into account the structural transformations during the **UOV**‐to‐IPC‐12 rearrangement (Figures 1 and 4), the pore system is changed from two dimensional (12+8)×10 to 1D (12+8) and should therefore be accompanied by the significant loss in microporosity caused by the disappearance of the interlayer porosity (due to the removal of d4rs the 10‐ring channels become 6‐rings, which are too small to be classed as pores). As expected, as measured using argon adsorption experiments (Supporting Information, Figure SI‐4), the micropore volume, *V*_micro_, for IPC‐12 material decreased to 0.052 cm^3^ g^−1^ from 0.111 cm^3^ g^−1^ in the parent **UOV** zeolite.

Given that the structural model is consistent with the XRD, the TEM and the adsorption measurements we are extremely confident that the predicted model correctly describes the connectivity in the IPC‐12 structure.

The results reported here are significant in that they illustrate that the ADOR process is not limited to one parent zeolite only. The further development of the ADOR technique, aiming towards the design of new **UOV**‐derived zeolites as analogues of the isoreticular zeolites IPC‐2, IPC‐6, IPC‐7, IPC‐9, and IPC‐10 that can be prepared from **UTL**, as well as the use of other parent zeolites is in progress. However, it is clear that each parent zeolite has subtly different behavior in the ADOR process and that there is still much to do to fully understand the key features of ADORable materials. Of particular note in this work was the worry that porous layers, such as those in the **UOV** topology (with perpendicular pores) would be unstable under the ADOR conditions. This worry has proved to be unfounded.

## Experimental Section

Materials, methods, and further characterization can be found in the Supporting Information. The research data supporting this publication can be accessed at https://doi.org/10.17630/4d5783d8‐493d‐43f4‐abd9‐39cf20c3dcf5.

## Conflict of interest

The authors declare no conflict of interest.

## Supporting information

As a service to our authors and readers, this journal provides supporting information supplied by the authors. Such materials are peer reviewed and may be re‐organized for online delivery, but are not copy‐edited or typeset. Technical support issues arising from supporting information (other than missing files) should be addressed to the authors.

Supplementary
