# Supplementary material for: Expansion of the ADOR Strategy for the Synthesis of Zeolites: The Synthesis of IPC‐12 from Zeolite UOV
Source: Angew Chem Int Ed Engl. 2017 Mar 13;56(15):4324–7. doi: 10.1002/anie.201700590 (PMC5396290; doi:10.1002/anie.201700590)
Supplement: Supplementary file 1 — Supplementary [file ANIE-56-4324-s001.pdf]

Angewandte Chemie
Eine Zeitschrift der Gesellschaft Deutscher Chemiker

Supporting Information

Expansion of the ADOR Strategy for the Synthesis of Zeolites: The Synthesis of IPC-12 from Zeolite UOV

Valeryia Kasneryk, Mariya Shamzhy, Maksym Opanasenko, Paul S. Wheatley, Samuel A. Morris, Samantha E. Russell, Alvaro Mayoral, Michal Trachta, Jiří Čejka, and Russell E. Morris**

anie_201700590_sm_miscellaneous_information.pdf

Supporting information

Materials Decamethonium bromide (>98.0%, TCI), germanium oxide (99.99 %, Sigma Aldrich), Cab-O-Sil M-5 (Supelco Analytical), hydrochloric acid (37%, Sigma Aldrich), acetic acid (99%, Lachner), orthophosphoric acid (85%, Fluka),aluminium nitrate nonahydrate (99.4%, Lachner), methanol (99.98%, Lachner), acetone (99.97%, Lachner) were used for the synthesis of zeolite samples and their treatments. Hydrofluoric acid (48 wt. % in H_2O, ANALPURE®), nitric acid (67 wt. % in H_2O, ANALPURE®), hydrochloric (36 wt. % in H_2O, ANALPURE®) supplied by Analytika spol. s.r.o. boric acid (>99.5%, Sigma) were used for decomposition of zeolites.

Syntheses of UOV zeolites Germanosilicate UOV samples were obtained from reaction mixtures with the following composition: 0.33 SiO_2 : 0.66 GeO_2 : 0.25 DMDH : 10 H_2O, using decamethonium dihydroxide (DMDH) as the structure-directing agent (SDA). Decamethonium dihydroxide was prepared from the bromide form by ion exchange using Ambersep® 900(OH) anion exchange resin (0.8 mmol of SDA per 1 g of anion exchange resin). The solution of DMDH was concentrated under low pressure (25 Torr) at 30 °C until the hydroxide concentration grown to > 1.5 mol/l. For UOV synthesis, certain amount of germanium oxide was dissolved in a mixture of water and DMDH. Silica (Cab-O-Sil M5) was added to the solution, and the mixture was stirred at room temperature for 30 min. After that, the reaction gels were autoclaved at 175 °C for 7-14 days under static conditions. The solid product was recovered by centrifugation, washed out several times with a copious amount of distilled water, dried at 65 °C during 12 h and finally calcined at 550 °C for 6 h with a temperature ramp of 2 °C/min under air flow (200 ml/min).

ADOR protocol 0.1 g of calcined samples of UOV was treated by 10 ml of selected treatment solutions (01.M, 1M, 4M and 12M HCl) at room temperature for 12 h. The produced solids were isolated by centrifugation, washed with methanol and acetone, dried at room temperature overnight. The obtained solids were calcined at 550 °C for 6 h with a temperature ramp of 2 °C/min.

Rietveld refinement Synchrotron X-ray diffraction data were collected on beamline I11 at the Diamond Light Source, UK. The wavelength of the radiation was 0.82604 Å. Rietveld refinement of the computationally-derived model for IPC-12 against this data converged satisfactory to R_{F2} = 0.1086, wRp = 0.0259 and Rp = 0.0189 using the GSAS suite of refinement programs. Bond distance restraints of Si-O, O-O and Si-Si of 1.61, 2.63 and 3.07 Å, respectively, with a weighting factor of 100 in GSAS. The isotropic displacement parameters (Uiso) were fixed at 0.04 and 0.05 for Si and O, respectively. The structural model refinement then converged to a reasonably chemically sensible structure. Fractional coordinates for the final model can be found in the supplementary material.

Characterization The structure and crystallinity of zeolites under study were determined by X-ray powder diffraction (XRD) using a Bruker AXS-D8 Advance diffractometer with a graphite monochromator and a position sensitive detector (Våntec-1) using CuKα radiation in Bragg-Brentano geometry at a scan rate of 0.25° (2θ/min). The chemical compositions of the samples were determined by an ICP/OES (ThermoScientific iCAP 7000) analysis. 50 mg of zeolite were mineralized in a mixture of 2 ml of HF, 4 ml of HNO_3, and 4 ml of HCl in the microwave. After cooling, the HF excess was eliminated by the complexation with the 15 ml of saturated solution of H_3BO_3 and final mixture was treated in microwave again. Then solutions under analysis were collected and diluted by ultrapure water to total volume of 250 ml.

The size and shape of UOV crystals were studied by a scanning electron microscopy (SEM, JEOL JSM-5500LV microscope). For the measurement the crystals were coated with a thin layer of platinum (~ 10 nm) in a BAL-TEC SCD-050 instrument.

Argon adsorption/desorption isotherms were measured using an ASAP 2020 (Micromeritics) static volumetric apparatus at liquid argon temperature (–186 °C). Prior to the sorption measurements, all samples were degassed with a turbomolecular pump at 300 °C for 8 h. The t-plot method was applied to determine the volume of micropores (V_{micro}).

The ^{19}F Solid-state MAS NMR spectra were acquired using a Bruker Advance III 600 MHz spectrometer equipped with a wide bore 14.1 T magnet. 0.1 g of zeolite was ground in the

mortar with 0.1 g of NH_4F. Samples were loaded into 2.5 mm rotors and rotated at MAS rates of 25 kHz. The chemical shift scale was shown relative to CCl_3F.

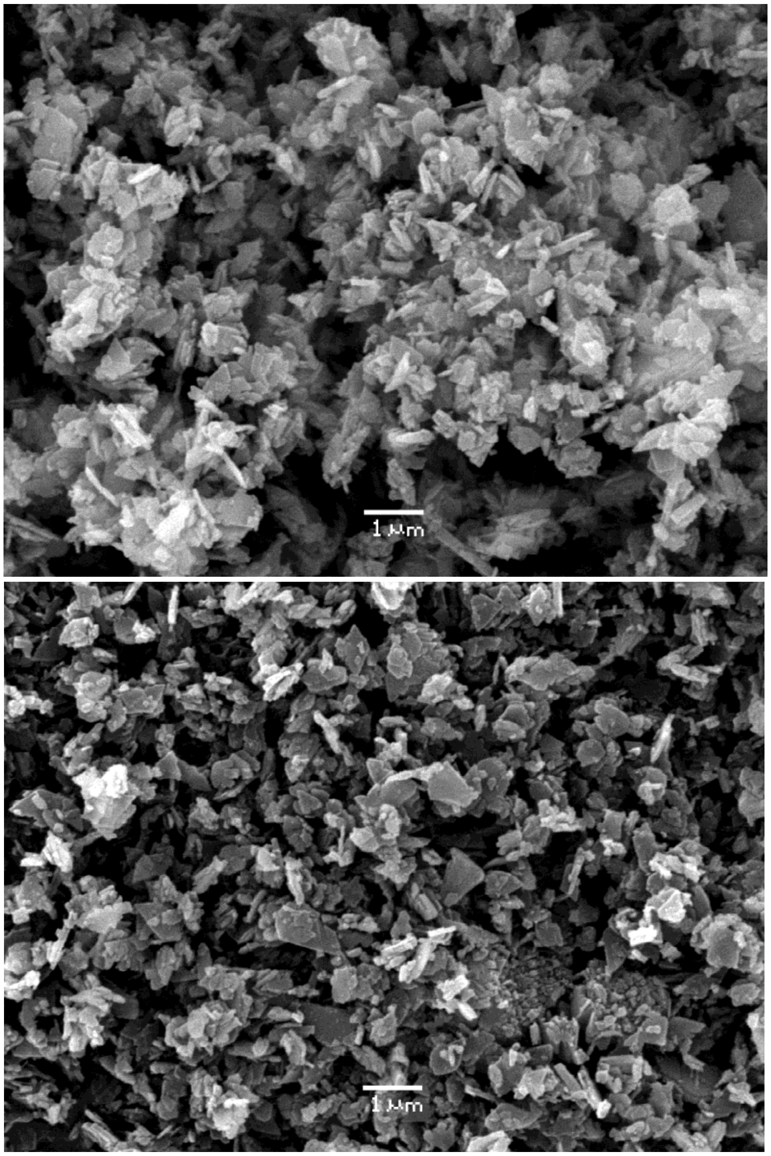

Figure SI-1. SEM images of the starting UOV zeolite sample used in the experiments (top) compared with an image of the final IPC-12 material (bottom).

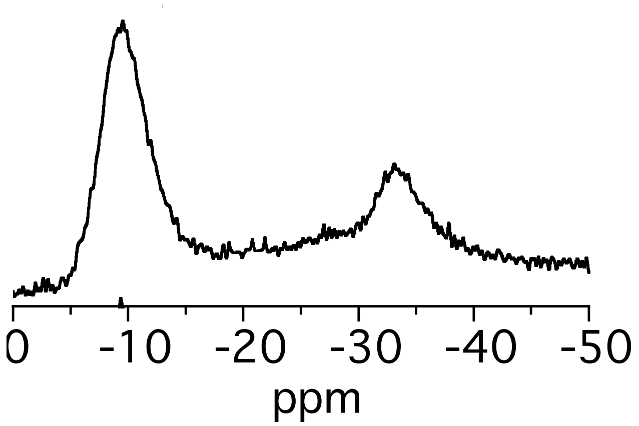

Figure SI-2 ^{19}F NMR spectrum of the starting UOV sample. The peak at around -10 ppm is typically assigned to F^- occluded in Ge_4Si_4 d4rs and a resonance at -30 ppm that is typically assigned to F^- located in the siliceous layer surrounded only by silicon atoms (See reference 12 in the main paper).

Table SI-1 Fractional coordinates of IPC-12 from the Rietveld refinement experiment. Unit cell parameter *a* = 7.511(11) Å, *b* = 21.638(4) Å, *c* = 37.736(29) Å, spacegroup A m m 2

Atom	x	y	z
Si1	0.0	0.0704(4)	0.2527(7)
Si2	0.3010(11)	0.1199(9)	-0.0016(5)
Si3	0.2027(11)	0.2489(10)	-0.0229(4)
Si4	0.2029(11)	0.2586(11)	-0.1009(4)
Si5	0.3019(10)	0.3554(9)	0.0277(5)
Si6	0.0	0.42783(34)	0.1295(7)
Si7	0.0	0.4289(4)	0.0528(7)
Si8	0.3018(11)	0.3461(8)	0.1529(5)
Si9	0.2052(11)	0.2727(8)	0.0886(6)
Si10	0.2067(11)	0.1373(10)	0.0762(4)
Si11	0.2030(13)	0.07231(33)	0.1441(6)
Si12	0.2986(11)	0.1412(10)	0.2123(5)
Si13	0.2042(11)	0.2779(11)	0.2183(4)
Si14	0.2039(11)	0.2673(12)	0.2971(4)
Si15	0.3039(11)	0.3514(8)	0.3578(5)
Si16	0.0	0.42824(35)	0.3695(6)
Si17	0.3034(11)	0.1576(8)	0.3390(5)
Si18	0.0	0.07265(34)	-0.0524(5)
Si19	0.0	0.0697(4)	0.3368(7)
O1	0.0	0.0	-0.0584(10)
O2	0.0	0.1058(9)	-0.0917(5)
O3	0.1752(10)	0.0937(9)	-0.0314(5)
O4	0.231(4)	0.1080(13)	0.0379(4)
O5	0.5	0.0900(12)	-0.0052(10)
O6	0.3233(28)	0.1943(9)	-0.0070(9)
O7	0.0	0.2245(14)	-0.0275(9)
O8	0.277(4)	0.2691(15)	-0.0614(4)
O9	0.2067(29)	0.3078(12)	0.0020(7)

O10	0.0	0.2808(17)	-0.1043(12)
O11	0.216(4)	0.1852(10)	-0.1092(6)
O12	0.3277(23)	0.2942(11)	-0.1286(5)
O13	0.5	0.3737(15)	0.0149(7)
O14	0.1774(10)	0.4156(8)	0.0306(7)
O15	0.3151(26)	0.3245(11)	0.0668(6)
O16	0.0	0.3924(7)	0.0908(7)
O17	0.0	0.5	0.0636(11)
O18	0.0	0.5	0.1231(11)
O19	0.1757(10)	0.4058(7)	0.1503(7)
O20	0.3180(30)	0.3243(12)	0.1937(5)
O21	0.5	0.3625(15)	0.1387(7)
O22	0.223(4)	0.2893(10)	0.1300(6)
O23	0.0	0.2732(10)	0.0769(10)
O24	0.2918(22)	0.2058(8)	0.0799(9)
O25	0.2929(28)	0.0929(15)	0.1065(5)
O26	0.0	0.1437(18)	0.0858(9)
O27	0.0	0.0985(15)	0.1448(11)
O28	0.203(6)	0.0	0.1474(13)
O29	0.3147(27)	0.1036(16)	0.1756(6)
O30	0.2195(34)	0.2086(10)	0.2035(7)
O31	0.5	0.1486(14)	0.2280(7)
O32	0.1760(10)	0.1054(7)	0.2396(7)
O33	0.281(4)	0.2802(17)	0.2581(4)
O34	0.0	0.2999(16)	0.2185(12)
O35	0.221(4)	0.1942(12)	0.3057(6)
O36	0.3235(26)	0.3049(12)	0.3251(6)
O37	0.0	0.2884(18)	0.2997(12)
O38	0.1755(10)	0.4080(9)	0.3480(6)
O39	0.5	0.3758(16)	0.3689(9)
O40	0.0	0.5	0.3763(12)
O41	0.5	0.1316(14)	0.3290(9)
O42	0.1761(10)	0.1033(7)	0.3515(8)
O43	0.0	0.0680(17)	0.2949(7)
O44	0.0	0.0	0.3524(10)
O45	0.0	0.0	0.2376(10)

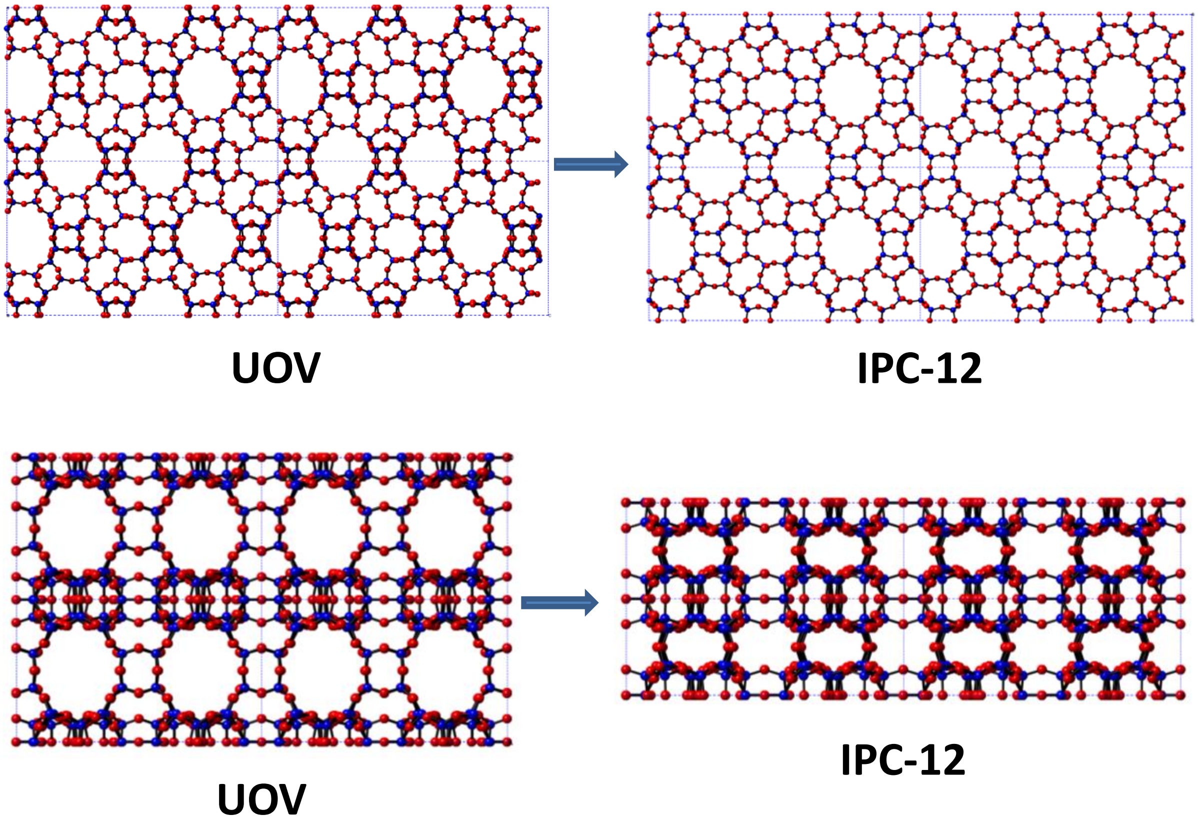

Figure SI-3. Schematic representation of UOV-to-IPC-12 structural transformation view from different projections (top – *bc*, bottom – *ab*)

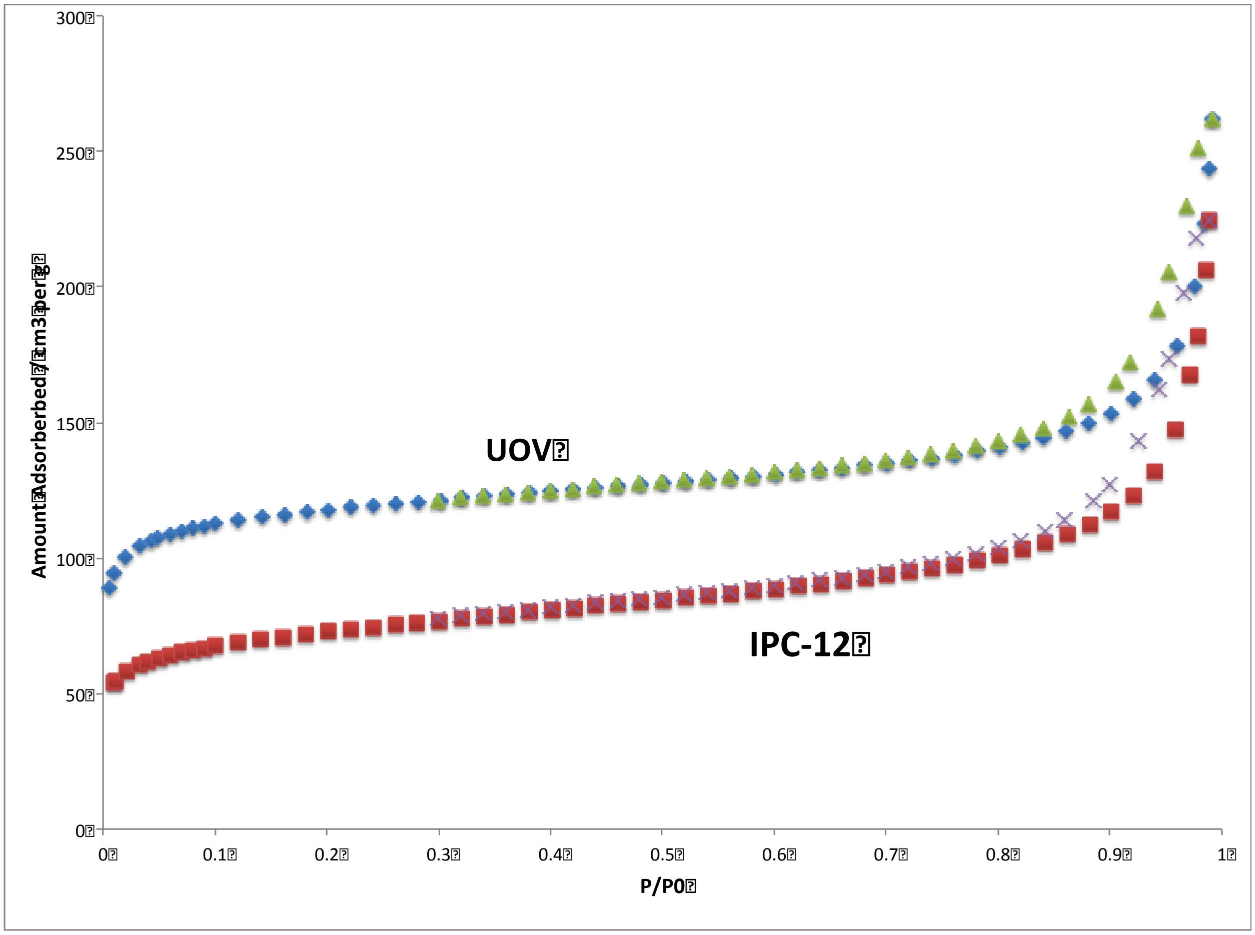

Figure SI-4 Argon isotherms for UOV and IPC-12 showing the expected reduction in adsorption capacity. Adsorption/desorption isotherms for UOV are shown in blue/green and for IPC-12 in red/purple.
